# Mathematical Proficiency in Adolescents with ASD

**DOI:** 10.1007/s10803-024-06645-3

**Published:** 2024-11-26

**Authors:** O. Cohen, N. Sukenik

**Affiliations:** Faculty of Education, Bar Ilan University, Ramat-Gan, Israel

**Keywords:** ASD, Adolescents, Mathematical proficiency, Procedural thinking, Arithmetic comprehension, Algebraic procedures

## Abstract

This study aimed to investigate the mathematical abilities of adolescents with Autism Spectrum Disorder (ASD) compared to typically developing (TD) peers, focusing on procedural thinking, arithmetic comprehension, and algebraic technique. Sixty-seven adolescents (31 with ASD, 36 TD) participated in the study. A comprehensive mathematics skills test, incorporating oral and written components, was individually administered to assess abilities across three main mathematical domains. The study employed a mixed-methods approach, combining quantitative analyses of group differences with qualitative assessments of response patterns. Significant differences were observed between ASD and TD groups across most mathematical measures, with TD adolescents generally outperforming those with ASD. Large effect sizes were noted in procedural thinking and algebraic procedures. However, no significant differences were found in word problem-solving. Within the ASD group, considerable variability was observed, with some individuals demonstrating age-appropriate mathematical abilities while others showed consistently low performance across all domains. The study highlights the complex nature of mathematical abilities in adolescents with ASD, characterized by significant group differences and within-group variability. These findings highlight the importance of individualized approaches in mathematical education for adolescents with ASD and emphasize the need for early identification and targeted interventions to address specific challenges in mathematical learning.

**Supplementary Information:**

The online version contains supplementary material available at 10.1007/s10803-024-06645-3.

## Introduction

Mathematics plays a foundational role in modern society, forming the basis of daily operations and influencing lifelong success and career development. However, its abstract nature poses particular challenges (Geary, 2004), especially for individuals with Autism Spectrum Disorder (ASD). The struggles experienced by individuals with ASD in grasping abstract mathematical concepts are often attributed to underlying issues with language comprehension (Bae, 2013). Unlike concrete, tangible concepts that can be readily observed and understood through sensory experiences, abstract mathematical notions rely heavily on language and symbolic representation (Mayes & Calhoun, 2003; Troyb et al., 2014). For individuals with ASD, who may encounter difficulties in processing and interpreting language, this reliance on linguistic comprehension can present substantial barriers to their mathematical learning and understanding. Furthermore, executive functioning deficits, particularly in working memory and flexibility, hinder their ability to process and manipulate numbers effectively (Russell, 1997). Attentional issues may prevent children with ASD from maintaining focus on overarching mathematical concepts (Courchesne et al., 2001) and visual-spatial processing challenges can impair their understanding of spatial relationships essential for geometry (Kuschner, Bodner, & Minshew, 2009). Furthermore, difficulties with generalization make it difficult for them to apply learned mathematical skills in new contexts (Minshew, Meyer, & Goldstein, 2002). Additionally, social communication difficulties can limit their ability to engage in the collaborative and communicative aspects of mathematical learning in classroom settings (Klin et al., 2002).

**Fig. 1:**
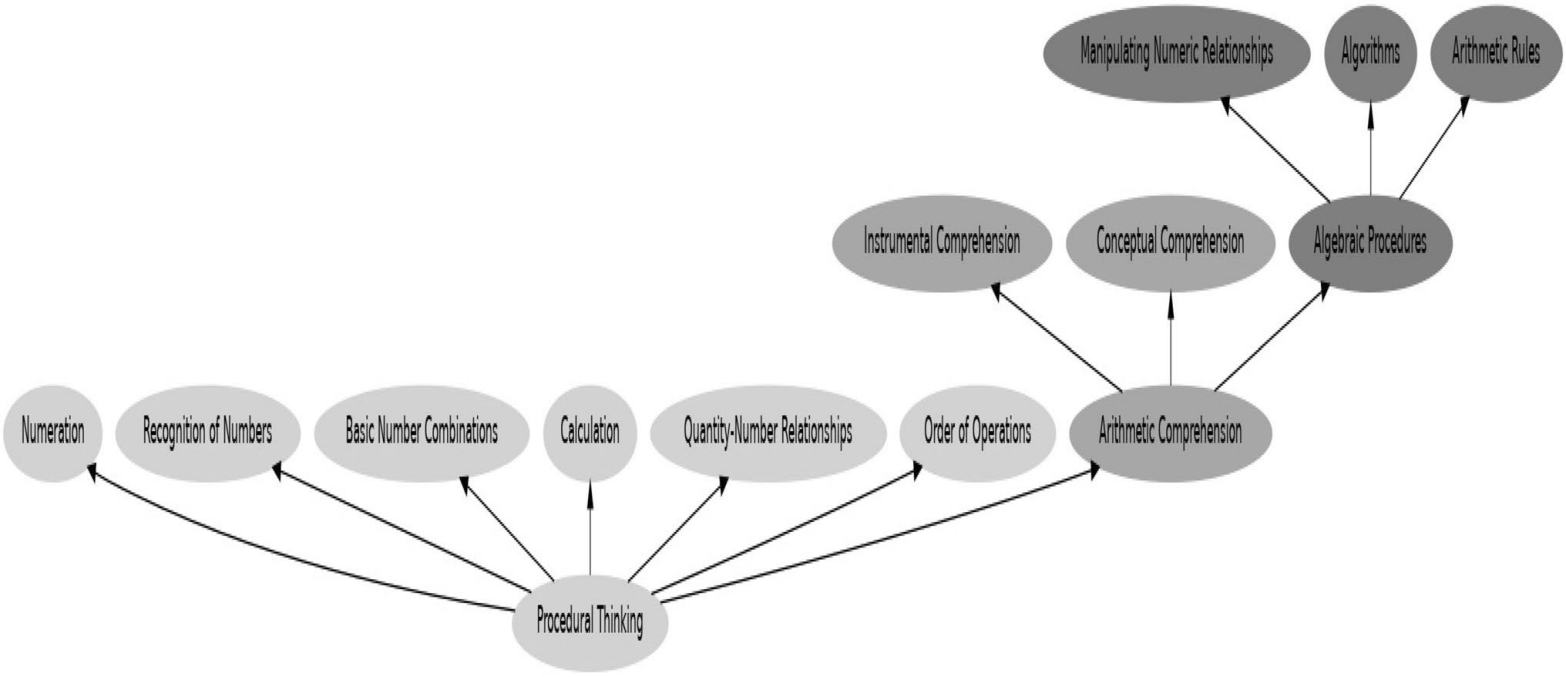
Mathematical domains in the current study

This study aims to investigate differences in mathematical abilities between adolescents with ASD and age-matched adolescents with TD. Guided by Siegler’s Integrated Theory of Numerical Development (2016), in our study, we have chosen to focus on procedural thinking, arithmetic comprehension, and algebraic procedures. This theory posits that numerical development occurs through a dynamic interplay between various cognitive processes and experiences across development. Procedural thinking, a foundational aspect of mathematics, involves fundamental cognitive processes such as numeration, calculation, and information processing. It serves as the bedrock upon which more advanced mathematical skills are built. Arithmetic comprehension encompasses both instrumental and conceptual understanding of mathematical principles, including problem-solving and the application of mathematical rules in diverse contexts. Finally, algebraic procedures represent the pinnacle of numerical development, involving symbolic representation, abstract reasoning, and the manipulation of mathematical structures. By focusing on these key areas, our study aims to provide a comprehensive understanding of the mathematical abilities of adolescents with ASD, emphasizing the importance of considering the dynamic and multifaceted nature of numerical development (Fig. 1).

Subsequent sections will examine each domain, portraying specific skills and emphasizing what has been found regarding the capabilities of children with ASD in these areas. This exploration aims to offer a comprehensive understanding of the mathematical challenges faced by individuals with ASD.

### Procedural Thinking

Procedural thinking, integral to mathematics, involves cognitive processes like numeration, calculation, and information processing (Singer et al., 2019). It serves as the foundation for arithmetic understanding (Jordan et al., 2009; Purpura et al., 2011). Within arithmetic, procedural thinking includes numeration, initiated around age two, involving generating sequential numbers through auditory perception and memory. Recognition of numbers, starting around ages 3–4, involves visual perception, initially focusing on numbers one through ten. Mastering basic number combinations, beginning around ages 4–5, involves children consolidating essential arithmetic facts for later recall (Costea et al., 2018). Understanding the quantity-number relationship, achieved around ages 4–5, signifies grasping that numbers represent specific quantities, crucial for tasks involving numerical sequences. As foundational processes develop (Geary, 2018), children acquire skills for fundamental arithmetic operations, understand the order of operations, and apply algorithmic thinking (Lee, 2022).

Given the essential role of procedural thinking in mathematical abilities (Siegler, 2016), it becomes imperative to explore this domain among students with ASD, as it illuminates their core challenges in mathematical learning. Baron-Cohen et al. (2001) explored the relationship between autistic characteristics and mathematical performance among students with ASD and no intellectual disability and found a non-linear correlation. Additionally, difficulties in procedural thinking are crucial in identifying learning disabilities in mathematics. Studies highlight vulnerabilities in arithmetic functioning among children diagnosed with ASD, with heightened co-occurrence rates between Mathematical Learning Disabilities (MLD) and ASD (Wei et al., 2015). Some researchers attribute the challenges that children with ASD face in procedural thinking to the Weak Central Coherence (WCC) theory (Graub, 2022), which is linked to verbal subitizing and complex information processing challenges. While procedural thinking in the autistic population is relatively unstudied, Titeca's study (2014) on 4–5-year-old children challenges conventional assumptions that procedural thinking abilities are impaired in children with ASD. The study revealed no significant differences in procedural thinking skills between children with ASD and typically developing peers. These disparate findings emphasize the necessity for additional investigations into the domain of procedural thinking abilities among individuals with ASD.

### Arithmetic Comprehension

Arithmetic comprehension involves the cognitive process of developing new procedures based on existing ones and utilizing analogical reasoning to draw parallels between familiar and novel systems with similar regularities (Barnett & Cleary, 2019). Arithmetic comprehension is categorized into Instrumental Comprehension, which emphasizes applying known mathematical rules, and Conceptual Comprehension, which delves into the 'why' behind procedures and understanding underlying principles. This extends beyond basic counting and calculation to encompass word problem-solving, estimation, numerical insight, and abstract estimation, particularly in mathematical word problems. Proficiency in solving these word problems demands skills in comprehending mathematical language, translating real-world issues into numerical representations, and understanding the effects and significance of arithmetic operations (Stillwell, 2019). Numerical insight, which includes estimation, a fundamental operation in arithmetic comprehension, involves visually comparing quantities, estimating without precise counting, and making educated guesses. This proficiency is integral for understanding mathematical concepts in a logical context, enabling individuals to solve word problems and appreciate the practical implications of mathematics in everyday life (Singer et al., 2019).

It is highly plausible to anticipate arithmetic comprehension challenges in children with ASD (Andersson & Ostergren, 2012; Geary et al., 2012). Titeca (2014) suggests a connection between the theory of "executive dysfunction" (Ozonoff et al., 1991) and arithmetic comprehension in children with ASD. Deficits in executive functions are considered potential contributors to Mathematical Learning Disabilities (MLD). These challenges in arithmetic comprehension may be attributed, in part, to verbal difficulties, especially in concept comprehension, understanding mathematical language, and solving word problems (Fleury et al., 2014; Wei et al., 2012). Language and communication deficits may initially impede students' understanding of mathematical concepts (Bae et al., 2015; Koyama et al., 2007; Whalon et al., 2020).

### Algebraic Technique

Algebra involves the symbolic representation of numerical expressions, marking a shift from concrete and numerical foundations to symbolic and abstract reasoning (Barnett & Cleary, 2015, 2019). This advanced thinking requires manipulating numerical relationships and mathematical structures, with proficiency depending on a solid arithmetic foundation. Mastery involves connecting knowledge of basic number combinations, mathematical laws, and symbols for both concrete and symbolic word problem-solving (Goya, 2023). Crucially, algebra shares algorithms and arithmetic rules, evident when solving word problems and equations. Understanding the " = " symbol as representation of equality is essential, and learners must grasp that symbols can denote variables, not just fixed numbers. Transitioning from unidirectional reading of equations to bidirectional information processing deepens learners' understanding of mathematical symbols and meanings, enabling them to engage in more complex word problem-solving and mathematical reasoning (Goya, 2023).

Concerns regarding the abilities in algebraic procedures of students with ASD are prevalent in the academic landscape. Approximately 25% of students diagnosed with ASD face challenges related to mathematic learning disabilities in general and in algebraic challenges specifically (Mayes & Calhoun, 2006). While they often exhibit proficient performance in algebraic procedures in the lower grades, where rote memorization is foundational, these students often encounter difficulties in algebra upon transitioning to middle school. The increasing abstraction and cognitive complexity of algebraic content, such as solving equations, understanding variables, and manipulating algebraic expressions, pose significant challenges. Students with ASD particularly struggle with word problem-solving, abstract reasoning, and the application of algebraic concepts to novel situations, which are identified as areas of weakness (Mayes & Calhoun, 2003; Whitby & Mancil, 2009). The shift towards these abstract concepts and the demand for advanced cognitive skills in algebra intensify existing challenges, emphasizing the need for targeted interventions and support mechanisms to facilitate students' mathematical development.

In summary, examining the mathematical abilities in children and adolescents with ASD reveals notable obstacles in procedural thinking, arithmetic understanding, and algebraic procedures. However, the intricacies of this field remain complex and not fully understood, marked by visible variations in mathematical abilities and a lack of comprehensive research.

### The Current Study

The aim of the current study was to investigate the mathematical abilities of adolescents with ASD in comparison to age-matched TD peers in three main mathematical areas: procedural thinking, arithmetic comprehension, and algebraic technique. Based on previous studies (Bae et al., 2015; Fleury et al., 2014; Troyb et al., 2014; Wei et al., 2012), we anticipated that TD adolescents would demonstrate better performance in mathematical domains, particularly in comprehending arithmetic concepts and utilizing algebraic techniques reliant on comprehension, compared to adolescents diagnosed with ASD. Given the known variability often found between participants diagnosed with ASD (Brynskov et al., 2017; Mayes & Calhoun, 2003; Titeca, et al., 2015), we hypothesized that a subset of adolescents diagnosed with ASD will demonstrate task performance commensurate with their chronological age, while others will exhibit performance lower than that of the control group. Furthermore, considerable heterogeneity was anticipated among adolescents with ASD across subtests within the same domain. Specifically, we anticipated that many of the adolescents with ASD would demonstrate proficiency in arithmetic procedures, yet encounter challenges with word problem-solving tasks (Baron-Cohen, et al., 2001; Troyb et al., 2014; Whalon et al., 2020).

## Method

### Participants

The study included 67 adolescent participants, 31 of whom had been diagnosed with Autism Spectrum Disorder (ASD) by qualified psychiatrists according to DSM guidelines prior to participation. All participants with ASD were enrolled in inclusive educational settings designed for adolescents with High-Functioning Autism (HFA), where they pursued an academic curriculum aligned with matriculation examinations. Consistent with the Ministry of Education’s requirements for special education, each participant with ASD had a recent and documented diagnosis (within the last three years). Under local legislation, a formal ASD diagnosis is mandatory for special education eligibility, reinforcing the validity of each participant’s diagnostic status. In Israel, ASD diagnoses are standardized according to DSM criteria, and terms such as High-Functioning Autism (HFA) and Low-Functioning Autism (LFA) are widely used to differentiate levels of functional independence. All participants in this study met the criteria for HFA.

Within the ASD cohort, the sex distribution included 24 males and 7 females, closely matching the typical male-to-female ratio of 4:1 reported in the literature (Potyrcha, 2022). Participants with ASD were between 12 and 19.2 years of age (*M* = 15.8 years, *SD* = 1.97 years) and were reported by their parents to have no additional known disabilities. Most participants with ASD demonstrated Nonverbal IQ (NVIQ) scores within the normal range, although six individuals scored below 80. These lower NVIQ scores may reflect task fatigue, as error rates increased towards the end of the task. To ensure linguistic consistency, only native Hebrew-speaking adolescents were included in the study.

The typically developing (TD) group comprised of 36 adolescents (Males: 27, Females: 9) matched in chronological age to the ASD group (*M* = 15.4 years, *SD* = 1.56 years) and with no known neurological impairments. Table 1 presents demographic variables, showing no significant differences in demographic variables between the two groups.

**Table 1:** Demographic characteristics for ASD and TD groups

	ASD		TD			
	(*n* = 31)		(*n* = 36)			*d*
Sex					χ^2^(1, *N* = 67) = 0.045, *p* = 0.05	
Male	24	(77.42%)	27	(75%)		
Female	7	(22.58%)	9	(25%)		
Age years *M* (*SD*) Y	15.8	(1.97)	15.4	(1.56)	*t*(65) = .804, *p* = 0.425	0.22
NVIQ *M (SD)*	94.09	(15.3)	100.22	(17.7)	*t*(65) = 1.511, *p* = 0.14	-0.36
Receptive vocabulary* M (SD)*	89.23	(14.37)	90.92	(12.79)	*t*(65) = 0.502, *p* = 0.587	-0.12
Linguistic variables						
Phonology	32.76	(45.16)	54.4	(33.83)	*t*(65) = 1.43, *p* = 0.16	-0.54
Morphology	74.41	(18.47)	83.52	(14.21)	*t*(65) = 2.27, *p* = 0.03*	-0.55
Syntax	68.4	(23.15)	64.52	(25.10)	*t*(65) = 0.65, *p* = 0.52	0.16
Pragmatics	246.29	(17.59)	296.08	(4.59)	*t*(65) = 0.23, *p* = 0.00*	-4.01
Semantics	94	(4.35)	92.44	(5.12)	*t*(65) = 0.88, *p* = 0.10	0.32

Note. *p < .05. **p < .01. ***p < .001. Cohen's d; small d > 0.2; medium d < 0.5; large d > 0.8

The provided dataset was a part of a larger study conducted at Bar Ilan University, encompassing various demographic and linguistic factors. Examining Table 1 reveals that distinctions in linguistic abilities were noticeable only in pragmatic and morphological tasks. Small effect sizes were observed for receptive vocabulary, semantics, and syntax, while age and NVIQ indicated medium effect sizes. Phonology, morphology, and pragmatics exhibited large effect sizes, indicating significant disparities between the groups. These results highlight notable variability, especially in pragmatic abilities, between adolescents with ASD and their typically developing peers.

### Tasks

#### Demographic Variables

*Receptive vocabulary* – Peabody Picture Vocabulary Test PPVT-5 (Dunn & Dunn, 2019) The PPVT 5 was used in its adapted Hebrew version (Isman et al., 2020).

The purpose of the test was to assess general vocabulary comprehension through picture recognition by pointing. The test contained 110 items ranked by difficulty level. Each item had four pictures. The researcher read a stimulus word, and the participant had to identify the most suitable picture among the four pictures. A correct answer earned one point, and an incorrect answer earned zero points. This test was previously administered to children with ASD and was found to be effective in assessing vocabulary comprehension (Brady, 2014). During data analysis, standard scores were used as per test guidelines.

### *Non Verbal IQ—*Raven's Progressive Matrices (Raven, 1998)

The test was administered to assess non-verbal cognitive levels (Raven, 1998) and to evaluate thinking processes from the simplest level to analogical thinking. The test consisted of abstract matching visual tasks. For each task, the subject was asked to find the appropriate item from the proposed distractors to complete the matrix. A correct answer earned one point, and an incorrect answer earned zero points. The possible raw score range was 0–60. During data analysis, raw scores were converted to standard scores based on Israeli norms (Israeli norms and guidelines—Chamberlain, 2008). This test was previously administered to children with ASD and was found to be effective in examining non-verbal cognitive abilities (Leytham, 2015).

#### *Linguistic Abilities Skills Tasks*

See Appendix A, which details the linguistic tasks that tested the following areas: Phonology, Morphology, Syntax, Pragmatics and Semantics.

#### *Mathematics Skills Test* (Sanduke Kachalon, 2021)

The original tool was taken from an MA thesis by Stav Sanduke Kachlon at Bar Ilan University, exploring the correlation between linguistic skills and arithmetic performance. For the current study, a section focusing on algebraic techniques was added, tailored to the eighth-grade curriculum. The Mathematics Skills Test aims to evaluate the participants' mathematical proficiency in three main areas: procedural thinking, arithmetic comprehension, and algebraic techniques. As each section included many subsections, a detailed overview of the tasks can be found in Appendix B. Participants underwent a series of mathematical questions presented both orally and in writing, requiring independent responses. Each participant was assigned a composite score, with a maximum of 37 points, which included the weighting of twenty dependent variables, distributed across procedural thinking (up to 23 points), arithmetic comprehension (up to 10 points), and algebraic techniques (up to 4 points). Scores were converted to percentages for statistical analysis. Additionally, each participant received scores in each tested mathematical sub-category (see Appendix B for examples and elaborate coding scheme).

In the initial phase of our research, we examined the distinctions between adolescents with ASD and TD through a comprehensive analysis of composite scores. This analysis involved the weighting of twenty dependent variables. Subsequently, to gain a deeper statistical understanding, we conducted analyses on the composite scores of each sub-domain, specifically examining the mastery of basic number combinations (a component of procedural thinking) and the ability to solve word problems (a facet of arithmetic comprehension). This task had not been previously administered to children with ASD. A pilot study was conducted before the current study to ensure reliability and validity. The pilot study data was excluded from the current analysis.

### Procedure

The research proposal was approved by the university's IRB committee and the Office of the Chief Scientist at the Ministry of Education (Approval Number 12791). With the necessary approvals secured, the researcher proceeded to seek permission from the principal of an inclusive school to conduct a pilot study, which included ten adolescents diagnosed with ASD. To conduct the broader research, the researcher contacted two additional school principals—one from a regular high school and the other from an inclusive high school for children with ASD. Upon obtaining consent, 80 letters explaining the study's purpose were distributed to parents through class teachers, resulting in 37 approvals from the inclusive school and 40 from the regular school.

Once parental approval was obtained, the research tasks were administered individually to each participant. Notably, six participants with ASD and four participants with TD withdrew from participation at various stages due to parental refusal (4), scheduling conflicts (3), and incomplete task performance (3). At each stage, participants attended one to two meetings, with the timing and frequency tailored to their individual abilities.

Data analysis for each research task adhered to the guidelines outlined in the original research tool. A comprehensive quantitative analysis was undertaken, encompassing the tallying of correct answers, determination of group averages, and calculation of standard deviations. To ensure accuracy, coding and transcriptions underwent evaluation by two experts proficient in language and mathematics. The judges' level of agreement was systematically assessed, with any discrepancies resolved through discussion until consensus was achieved.

At the group level, means and standard deviations were calculated and a Shapiro–Wilk test was conducted to assess the normality of each variable in the dataset. The results indicated that none of the variables followed a normal distribution, as all *p*-values were below the threshold of 0.05.

Therefore in order to test for potential disparities in mathematical skills between adolescents with ASD and TD children a Mann Whitney test was performed. Correlations between mathematical variables and background variables in each of the subgroups utilized a Spearman correlation analysis as data was not normally distributed and subgroup sizes were small.

To explore potential subgroups among adolescents diagnosed with ASD exhibiting comparable proficiency levels in mathematics, individual profiles were created. The analysis employed the specialized 'SINGLIMS' software, developed by Crawford and Garthwaite in 2012. This software, an abbreviation for "Single-Case Linear Models," is tailored to aid statistical analysis in single-case experimental designs (SCEDs), commonly utilized in applied psychology and related fields to assess the impact of interventions on individual participants. In our study, 'SINGLIMS' established a performance threshold for each mathematical measure, allowing comparison of individual scores with those of a control group. This comparison aimed to determine whether specific scores indicated intact or impaired mathematical abilities. Furthermore, drawing from the Single-Case Methodology in Neuropsychology (Crawford & Howell, 1998) we sought to describe each ASD participant's mathematical performance relative to a carefully matched control sample. This comparison involved assessing each ASD participant's mathematical performance against the mean and standard deviation of a control group comprising typically developing adolescents matched for age (TD). These analyses provided insight into individual mathematical profiles, highlighting distinctions between age-matched participants and those underperforming relative to the control group. For tasks where the control group performed at ceiling (resulting in no variation and preventing the use of t-tests to compare the groups), an impairment threshold was set at 90%. In this context, ASD individuals scoring 90% correct or higher were considered to have unimpaired performance, even if their scores were lower than those of the TD group. This approach follows the recommendation by Willmes (1990), highlighting the necessity of establishing an arbitrary cutoff in such cases. Subsequently, distinct subgroups of adolescents with ASD were identified based on similar performance patterns.

Regarding the formation of subgroups based on the individual profiles, we employed a methodical approach based on three distinct profiles. First, Profile one encompassed individuals whose performance ranged from those closely matching the TD group in each subdomain to those with low performance across up to three subdomains, and whose low scores could be traced to behavioral or attention issues. Profile two included individuals with low performance between 4 to 6 subdomains compared to the TD group. This profile sought to identify individuals exhibiting moderate levels of underperformance across multiple areas. Finally, Profile three comprised individuals with low performance in seven or more subdomains compared to the TD group, hence showing pervasive difficulties across a wide range of mathematical domains. By delineating these distinct profiles, we aimed to provide a nuanced understanding of the heterogeneity in mathematical abilities among adolescents with ASD. For each subgroup, due to the small amount of participants, a Spearman correlation was conducted between mathematical variables and background variables.

## Results

Initially, a comparison was made between adolescents with ASD and TD by accumulating all the metrics within each sub-domain tested (see appendix B) and subsequently conducting the comparison. Mann–Whitney tests were conducted to assess potential differences in mathematical abilities within procedural thinking, arithmetic comprehension, and algebraic techniques between the groups. Table 2 presents the composite scores of mathematical variables for each study group (ASD, TD).

**Table 2:** Group differences in composite mathematical measures, for ASD and TD Adolescents

	ASD	TD	*U*	*p*	*r*
	*M* (*SD*)	*M (SD)*			
Mathematical scores	51.96 (27.09)	75.23 (22.95)	267.5	0.000***	0.44
Procedural thinking	47.97 (27.30)	72.58 (22.02)	261.5	0.000***	0.45
Mastering the basic number combinations	45.16 (29.7)	77.78 (21.15)	213	0.000***	0.53
Arithmetic comprehension	77.0 (29.82)	82.22 (27.68)	0.00	0.005**	0.33
Word problems	70.97 (31.98)	83.56 (30.83)	0.17	0.17	0.13
Algebraic technique	37.9 (42.27)	72.92 (32.39)	0.00	0.000***	0.41

Note. *p < .05. **p < .01. ***p < .001

In the next step, the skills that were tested were: mastering the basic number combinations (a component of procedural thinking) and solving word problems (a part of arithmetic comprehension). Significant differences were identified between the ASD and TD groups in the composite variables of Mathematical scores, Procedural thinking, Mastering basic number combinations and Algebraic technique (*p* < 0.000) and in Arithmetic comprehension (*p* = 0.005) in favor of the TD group. Only in one variable, Word problems, no significant differences were observed between the two groups but it should be noted that the TD group mean was higher. Most variables showed medium to large effect sizes, suggesting notable differences in mathematical abilities between adolescents with ASD and TD peers.

Given the significant differences as well as the large standard deviations in both groups regarding the composite mathematical scores, a more fine-grained examination was needed for each of the three mathematical domains separately.

### Procedural Thinking

The first focus was on procedural thinking, subdivided into two components: basic number combinations and algorithms. In the first segment, we assessed the differences between the two groups in mastering basic number combinations. This phase involved oral testing, with participants required to promptly respond to questions, aiming for both speed and accuracy.

As seen in Table 3, The Mann–Whitney tests indicated that TD adolescents outperformed those with ASD across all Procedural Thinking basic number combinations measures. Significant differences were found in "Sums to 10 " (*U* = 398.0, *p* < 0.01) with a medium effect size (*r* = 0.25), as well as in "Sums to 20" (*U* = 841.0, *p* < 0.001) with a large effect size (*r* = 0.43). Similarly, TD adolescents showed superior performance in "Multiplication" (*U* = 257.5, *p* < 0.001) with a large effect size (*r* = 0.46), and in "Division" (*U* = 240.0, *p* < 0.001) with a medium effect size (*r* = 0.48). These findings suggest that TD adolescents demonstrate significantly higher proficiency in Procedural Thinking basic number combinations tasks compared to their peers with ASD, with effect sizes ranging from medium to large.

**Table 3:** ASD/ TD group differences in procedural thinking basic number combinations

	ASD	TD			
	M (SD	M (SD)	U	p	r
Sums to 10	72.58 (36.15)	91.67 (22.36)	398	0.01**	0.24
Sums to 20	45.16(37.32)	77.78 (27.89)	841	0.000***	0.43
Multiplication	32.26(33.04)	69.44 (32.24)	257.5	0.000***	0.46
Division	30.65(35.77)	72.22 (32.61)	240	0.000***	0.48

Note. *p < .05. **p < .01. ***p < .001

Table 4 presents the analysis of procedural thinking variables related to algorithmic procedures. The Mann–Whitney analysis showed that TD adolescents outperformed those with ASD across several algorithmic procedures, including "Subtraction using written algorithms," "Multiplication using written algorithms," with significant differences (all *p* < 0.01) and "Long-division," with significant differences (all *p* < 0.05) and effect sizes ranging from medium to large. However, the ASD group demonstrated better performance than the TD group on the "Order of operations" task (*U* = 701.0, *p* < 0.05, *r* = 0.21), with a small effect size. These results indicate that while TD adolescents generally have greater proficiency in algorithmic procedures, adolescents with ASD may show relative strengths in certain areas, such as carrying out the order of operations.

**Table 4:** ASD / TD group differences in procedural thinking—algorithmic procedures

	ASD	TD	*U*	*p*	*r*
	*M(SD)*	*M(SD)*			
Producing a sequential counting strategy	67.74 (39.89)	80.56 (34.39)	459	0.14	0.15
Addition- written algorithm	61.29 (49.51)	77.78 (42.16)	466	0.14	0.14
Subtraction- written algorithm	31.18 (37.45)	53.7 (40.06)	387.5	0.02*	0.26
Combining procedures: addition and subtraction	67.74 (47.52)	77.78 (42.16)	502	0.36	0.08
Multiplication- written algorithm	48.92 (31.9)	73.61 (26.54)	309.5	0.002**	0.38
Long-division	38.71 (49.51)	63.89 (48.71)	417.5	0.04*	0.21
Order of operations	50 (38.73)	31.94 (24.36)	701	0.04*	0.21

Note. *p < .05. **p < .01. ***p < .001

### Arithmetic Comprehension

The second area tested was arithmetic comprehension, which required further investigation due to differences found in the previous stage. Table 5 presents the results of additional tests conducted on its three subparts. Significant differences between the ASD and TD groups were observed in Word problems that require metacognitive thinking and Numerical insight (p < 0.05), with the TD adolescents demonstrating higher abilities in this area. On Word problems that require process thinking, a significant difference was not found between the groups but it should be noted that the TD group mean was higher. The effect sizes indicated that the TD group generally outperformed the ASD group across all measures, with the largest differences noted in numerical insight and metacognitive thinking in word problems.

**Table 5:** ASD / TD group differences in arithmetic comprehension

	ASD	TD	*U*	*p*	*r*
	*M (SD)*	*M(SD)*			
Word problems that require process thinking	74.19 (38.45)	84.72 (33.42)	473	0.17	0.13
Word problems that require metacognitive thinking	67.74 (34.94)	82.41 (35.17)	405	0.02*	0.23
Numerical insight	62.58 (32.96)	81.11 (27.44)	368.5	0.01*	0.29

Note. *p < .05. **p < .01. ***p < .001

### Algebraic Technique

The third area tested was algebraic technique, which included two measures presented in Table 6. TD adolescents performed significantly better than those with ASD in algebraic procedure (p < 0.000) and solving a word problem with unknown numbers (p < 0.05), with particularly large differences noted in the procedure measure.

**Table 6:** ASD / TD Group Differences in Algebraic Technique

	ASD	TD	*U*	*p*	*r*
	*M (SD)*	*M (SD)*			
Procedure	45.16 (47.18)	86.11 (25.67)	297.5	0.000***	0.40
Solving a word problem with unknowns	33.87 (41.61)	59.72 (44.43)	384	0.019*	0.26

Note. *p < .05. **p < .01. ***p < .001

In conclusion, the two groups showed significant differences across most mathematical variables, with the TD group performing better overall. Notably, adolescents with ASD outperformed the TD group in only the order of operations variable. The effect sizes for these differences varied from small to large, underscoring considerable disparities in the majority of areas.

### Individual Profiles

Due to the large standard deviations observed in the current sample, it became necessary to analyze the results at an individual level. Each ASD participant's score on each component of the mathematical task was compared to the TD group using Crawford and Garthwaite's t-test (2012), as previously described. This analysis established a threshold distinguishing impaired from intact performance relative to the TD group. Subsequently, individual profiles were constructed, leading to the formation of subgroups among the adolescents with ASD. Table 7 presents the individual scores of the adolescents with ASD, split into three distinct subgroups: the upper section represents those displaying age-equivalent mathematical proficiency, the middle section represents those manifesting inconsistent mathematical abilities, and the lower section represents those presenting low scores in all fields tested.

**Table 7:**
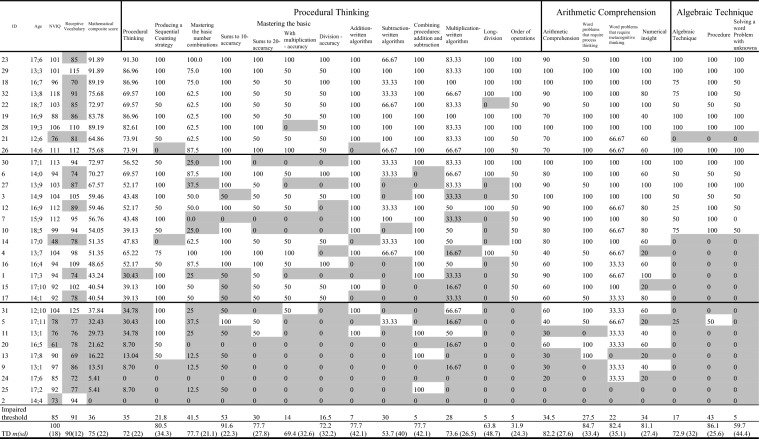
Individual profiles and subgroup division of the ASD group

Note. Cells painted in gray are cells in which the score was found to be significantly lower than the control group impaired threshold

As seen in Table 7, nine participants exhibited age-equivalent mathematical abilities, i.e., their mathematical abilities were not significantly different from those of the TD age-matched group. It should be noted that when assessing the mathematical abilities of participants with ASD, 'normal' performance was considered even in the presence of a single low score, attributed to factors such as lack of attention, fatigue, or refusal to participate towards the end of a session. This classification also included instances of confusion in writing figures or a singular action leading to a trailing error in the procedure.

Thirteen participants with ASD demonstrated uneven mathematical proficiency, with some scores not significantly different from those of the TD age-matched group, while others were significantly lower. Within this subgroup, a prevalent characteristic was the presence of low procedural thinking scores (such as reduced performance in measures of accuracy of retrieving basic mathematical concepts) and low achievements in algebraic techniques, although many demonstrated age-equivalent arithmetic comprehension scores (excluding two participants with low scores in estimation abilities-numerical insight). Finally, nine participants with ASD showed significantly lower scores than the TD age-matched group on all mathematical abilities tested.

We next examined whether any background variables were associated with specific profiles. Given that the data did not meet the normality assumptions required for parametric tests, we conducted a Spearman correlation analysis to explore the relationships among background variables and mathematical abilities within both the ASD and TD groups (see Table 8). In both groups, age showed no significant correlation with any other variable. However, strong correlations were observed between NVIQ and receptive vocabulary, as well as between NVIQ and all mathematical scores in both groups, suggesting a consistent association between cognitive abilities and mathematical performance across both groups. Mathematical sub-scores were also intercorrelated in both groups. Notably, a difference emerged between groups: in the ASD group, receptive vocabulary was not correlated with any mathematical variable, whereas in the TD group, receptive vocabulary was significantly correlated with all mathematical variables. This strong correlation in the TD group suggests a close relationship that may contribute to our understanding of the potential influence between linguistic and mathematical domains.

**Table 8:** Spearman correlation coefficients and significance levels for study variables in the ASD and TD groups

	Age	NVIQ	Receptive vocabulary	Mathematical scores	Procedural thinking	Arithmetic comprehension	Algebraic technique
Age		0.30 (*p* = 0.07)	-0.19 (*p* = 0.26)	0.14 (*p* = 0.40)	0.09 (*p* = 0.57)	0.13 (*p* = 0.42)	0.21 (*p* = 0.20)
NVIQ	-0.06 (*p* = 0.74)		0.42 (*p* = 0.01)*	0.71 (*p* = 0.00)*	0.69 (*p* = 0.00)*	0.57 (*p* = 0.00)*	0.63 (*p* = 0.00)*
Receptive vocabulary	-0.20 (*p* = 0.25)	0.57 (*p* = 0.001)*		0.41 (*p* = 0.01)*	0.38 (*p* = 0.02)*	0.39 (*p* = 0.01)*	0.36 (*p* = 0.02)*
Mathematical scores	0.02 (*p* = 0.90)	0.55 (*p* = 0.001)*	0.30 (*p* = 0.09)		0.94 (*p* = 0.00)*	0.78 (*p* = 0.00)*	0.83 (*p* = 0.00)*
Procedural thinking	-0.06 (*p* = 0.71)	0.44(*p* = 0.01)*	0.28 (*p* = 0.11)	0.95 (*p* = 0.00)*		0.62 (*p* = 0.00)*	0.68 (*p* = 0.00)*
Arithmetic comprehension	0.11 (*p* = 0.52)	0.60 (*p* = 0.00)*	0.27 (*p* = 0.12)	0.83 (*p* = 0.00)*	0.69 (*p* = 0.00)*		0.70 (*p* = 0.00)*
Algebraic technique	0.16 (*p* = 0.376)	0.58 (= 0.00)*	0.31 (*p* = 0.08)	0.85 (*p* = 0.00)*	0.71 (*p* = 0.00)*	0.72 (*p* = 0.00)*	

^*^p < 0.05

Note: ASD group correlations bottom, TD group correlations top

After looking at correlations at the group level we wanted to fine grain the analysis by looking at each subgroup of children with ASD. As the number of participants in each subgroup was relatively small, we used a Spearman’s Rank Correlation to test the connection between background variables and mathematical variables (Table 9). It should be noted that a Kruskal–Wallis test showed no significant difference in age between the three groups (*H* = 0.11 *p* = 0.95).

**Table 9:** Combined correlation table for the three ASD subgroups

Variable Pair	Group 1	Group 2	Group 3
Age – NVIQ	0.23 (*p* = 0.55)	-0.16 (*p* = 0.61)	-0.24 (*p* = 0.52)
Age – receptive vocabulary	-0.08 (*p* = 0.84)	0.04 (*p* = 0.90)	-0.66 (*p* = 0.05)
Age – Mathematical SCORES	0.24 (*p* = 0.54)	-0.26 (*p* = 0.39)	-0.17 (*p* = 0.66)
Age – procedural thinking	0.14 (*p* = 0.72)	-0.56 (*p* = 0.04)*	-0.24 (*p* = 0.54)
Age – arithmetic comprehension	0.26 (*p* = 0.49)	0.15 (*p* = 0.61)	-0.17 (*p* = 0.65)
Age – Algebraic Technique	0.29 (*p* = 0.44)	-0.05 (*p* = 0.88)	0.55 (*p* = 0.12)
NVIQ – Receptive Vocabulary	0.58 (*p* = 0.10)	0.30 (*p* = 0.31)	0.14 (*p* = 0.71)
NVIQ – mathematical scores	-0.02 (*p* = 0.96)	0.67 (*p* = 0.01)*	0.15 (*p* = 0.69)
NVIQ – procedural thinking	-0.50 (*p* = 0.17)	0.39 (*p* = 0.19)	0.27 (*p* = 0.49)
NVIQ – arithmetic comprehension	0.19 (*p* = 0.63)	0.59 (*p* = 0.033)*	0.03 (*p* = 0.94)
NVIQ – algebraic technique	0.22 (*p* = 0.56)	0.62 (*p* = 0.02)*	-0.14 (*p* = 0.72)
Receptive vocabulary – mathematical scores	0.28 (*p* = 0.47)	-0.10 (*p* = 0.73)	0.11 (*p* = 0.78)
Receptive vocabulary – procedural thinking	-0.07 (*p* = 0.85)	0.05 (*p* = 0.87)	-0.01 (*p* = 0.98)
Receptive vocabulary – arithmetic comprehension	0.11 (*p* = 0.78)	-0.12 (*p* = 0.69)	0.24 (*p* = 0.54)
Receptive vocabulary – algebraic technique	0.66 (*p* = 0.05)	-0.03 (*p* = 0.92)	-0.07 (*p* = 0.86)
Mathematical composite score – procedural thinking	0.83 (*p* = 0.00)*	0.65 (*p* = 0.01)*	0.90 (*p* = 0.00)*
Mathematical composite score – arithmetic comprehension	0.64 (*p* = 0.06)	0.66 (*p* = 0.01)*	0.88 (*p* = 0.00)*
Mathematical composite score – Algebraic Technique	0.72 (*p* = 0.02)*	0.85 (*p* = 0.00)*	0.41 (*p* = 0.27)
Procedural Thinking – Arithmetic Comprehension	0.27 (*p* = 0.48)	-0.02 (*p* = 0.95)	0.62 (*p* = 0.07)
Procedural thinking – algebraic technique	0.60 (*p* = 0.08)	0.28 (*p* = 0.35)	0.28 (*p* = 0.46)
Arithmetic comprehension – algebraic technique	0.15 (*p* = 0.71)	0.67 (*p* = 0.01)*	0.28 (*p* = 0.46)

signification for bold values ^*^p < 0.05

Note: Group 1 – age matched scores, Group 2 – uneven performance across tasks, Group 3 – overall low scores

As shown in Table 9, in the first group of adolescents with ASD, who had age-matched scores on most research variables, only two significant correlations were identified: one between Mathematical Scores and Procedural Thinking (*rs* = 0.52, *p* = 0.03), and another between Mathematical Scores and Algebraic Technique (*rs* = 0.49, *p* = 0.04). In contrast, Group 2, which demonstrated uneven task performance, had the most correlations. A significant negative correlation was found between Age and Procedural Thinking (*rs* =  − 0.56, *p* = 0.044), indicating that age alone does not predict task performance. Additionally, a significant positive correlation was observed between NVIQ and Mathematical Scores (*rs* = 0.67, *p* = 0.012), as well as with Arithmetic Comprehension (*rs* = 0.59, *p* = 0.033) and Algebraic Technique (*rs* = 0.62, *p* = 0.022), highlighting the role of NVIQ in supporting task performance. Furthermore, significant positive correlations were found between the Mathematical composite score and Procedural Thinking (*rs* = 0.65,* p* = 0.01), Arithmetic Comprehension (*rs* = 0.66, *p* = 0.01), and Algebraic Technique (*rs* = 0.85, *p* = 0.00). In Group 3, which exhibited overall low scores, there were strong significant correlations between Mathematical Scores and Procedural Thinking (*rs* = 0.90, *p* = 0.001) and Arithmetic Comprehension (*rs* = 0.88, *p* = 0.002). These strong correlations within Group 3 suggest that, despite lower overall scores, a shared underlying skill set may contribute to mathematical abilities.

## Discussion

The current study contributes to the understanding of mathematical proficiency in adolescents with ASD by examining three key domains: procedural thinking, arithmetic comprehension, and algebraic technique. Informed by the existing literature and previous research gaps, our investigation explores the distinct challenges faced by adolescents with ASD building upon the insights provided by Mayes and Calhoun (2003) and Bae et al. (2015).

Although demographic variables were carefully compared across the adolescent groups, revealing minimal differences in linguistic and cognitive abilities, significant differences emerged in mathematical abilities. This aligns with Mayes and Calhoun's (2003) research, which identified a mismatch between the cognitive abilities of adolescents with ASD and their mathematical achievements. These results support the assertion that a higher prevalence of mathematical difficulties may exist among children with ASD compared to the general population, as previously documented by various researchers (Jordan et al., 2009; Titeca et al., 2015; Fyfe et al., 2019; Zdziechowska-Dzierzgwa, et al., 2022).

In procedural thinking, adolescents with ASD showed significantly lower achievements compared to the control group across most measures. The ASD group outperformed the TD group only in the Order of operations variable. The ASD group had markedly lower abilities in almost all mastering the basic number combinations. They also performed worse in producing a sequential counting strategy, addition-written algorithm, and combining addition and subtraction procedures. These findings highlight the need for targeted support in procedural thinking skills to help adolescents with ASD improve their mathematical proficiency. Our findings resonate with Titeca's (2014) research, which found that primary school children with ASD perform significantly lower than their TD peers. Titeca's study examines the early numerical competencies and mathematical abilities in children with ASD, highlighting distinct patterns and challenges in their development of numerical skills that are markedly different from those observed in typically developing children. Our research extends these insights by examining how these early difficulties in numerical competencies may continue or change as children with ASD grow into adolescence. The consistency in findings across different age groups suggests that these mathematical challenges are enduring aspects of ASD, impacting individuals throughout their educational trajectories, but future longitudinal research is needed to fully understand these findings.

The practical implications of procedural difficulties in adolescents with ASD highlight the need for early interventions and ongoing support as they get older. Early interventions can help establish basic skills to manage procedural tasks more effectively. Continuous support is essential as these individuals transition to higher educational levels where mathematical content becomes increasingly complex. Tailored educational programs that focus on enhancing procedural skills, along with ongoing support, can help mitigate the long-term impact of these difficulties.

In the realm of arithmetic comprehension, TD adolescents showed higher numerical insights compared to their counterparts with ASD in word problems that require metacognitive thinking and numerical insight. However, no significant differences emerged in word problem-solving that require process thinking between the two groups, challenging previous findings (Bae et al., 2015; Fleury et al., 2014; Wei et al., 2012; Whalon et al., 2020). This contrasts with research on children aged 6–12 with ASD, where lower word problem-solving abilities were observed compared to their typically developing peers. Inconsistencies in outcomes may stem from two potential factors. First, while prior research has indicated weakened linguistic abilities in children with ASD, in the current study no differences were detected in linguistic abilities between the ASD and TD adolescent groups. This suggests that language proficiency may not have been the primary source of difficulty. Alternatively, differences may arise from differences in the content of verbal questions utilized in previous studies. Some questions in those studies required understanding the meaning of social situations (Koyama et al., 2007), often impaired in children with ASD. Our careful selection of verbal questions devoid of social context may contribute to the observed success of adolescents with ASD in these questions relative to previous studies, albeit still below their peers.

In the domain of algebraic technique, adolescents with typical development exhibited greater proficiency compared to their counterparts with ASD. This aligns with the reduced achievements observed in adolescents with ASD in the realm of procedural thinking, which is foundational to algebraic technique (Goya, 2023; Vukovic & Lesaux, 2013). Consequently, it is plausible to infer that the lower achievements in algebraic technique stem from challenges in mastering skills foundational to this domain. Further examinations within the field revealed outcomes consistent with those observed in our study, in the realm of algebra. These additional studies corroborate and reinforce the findings presented in our research (Mayes & Calhoun, 2003a; Whitby & Mancil, 2009).

The observed effect sizes provide valuable insights into the magnitude of differences between adolescents with ASD and their TD peers across various mathematical domains. The effect sizes ranged from small to large, indicating varying degrees of disparity. Notably, large effect sizes were observed in procedural thinking measures and the algebraic procedure measures, suggesting substantial gaps in these areas. These findings highlight the significant challenges that adolescents with ASD face in procedural thinking, which encompasses the ability to execute sequences of operations and apply systematic approaches to problem-solving.

Medium effect sizes were found in solving verbal problems with unknowns and numerical insight, highlighting significant but less pronounced differences. Small effect sizes in some arithmetic comprehension measures indicated minimal differences, suggesting that some adolescents with ASD can perform comparably to their peers in certain areas. These effect sizes underscore the importance of recognizing the specific mathematical challenges faced by adolescents with ASD, particularly in areas where the largest disparities were observed.

Due to the considerable variation observed within the adolescent group diagnosed with ASD across all researched domains, individualized profiles were constructed. These profiles showed areas of strength and weakness for each participant with ASD as compared to the age-matched TD group performance. Three distinct profiles emerged with sub-group sizes varying from 9 to 13 participants. The findings suggest a lack of a completely homogeneous profile, aligning with similar observations in other studies within the field that reported substantial heterogeneity in ASD participant abilities (Baron-Cohen et al., 2001; Wei et al., 2015).The most prevalent profile identified was one of unevenness. This implies that individuals with ASD may exhibit good proficiency in certain mathematical skills while displaying lesser proficiency in other skills within the same domain.

Upon analyzing behavioral patterns within the ASD group, we identified several trends. Many participants showed difficulties in accuracy of recalling basic concepts, which aligns with Graub's, 2022 study. This study noted significant procedural thinking skill disparities between children with ASD and their TD peers. Typically, those with uneven profiles displayed weaknesses in procedural thinking, yet their arithmetic comprehension skills were comparable to age matched TD adolescents. The emergence of consistent patterns among adolescents sharing similar profiles suggests potential external influences, such as specialized educational interventions or curricula focusing on specific mathematical domains. Future research might explore how different educational settings and curricula affect the mathematical abilities of adolescents with ASD.

Moreover, although not tested in the current study, working memory capacities should be considered as a complementary explanation for the observed differences in mathematical abilities. Working memory is crucial for storing and manipulating information over short periods, which is essential for solving mathematical problems that involve multiple steps and complex procedures (Alloway, 2009). Deficits in working memory, commonly observed in individuals with ASD, can hinder their ability to perform calculations, follow multi-step instructions, and maintain focus on tasks. These cognitive challenges can result in difficulties with procedural thinking, mastering basic number combinations, and solving word problems, all of which were areas where adolescents with ASD showed significantly lower performance compared to their TD peers.

It is noteworthy that, although they are not the majority, several adolescents with ASD achieved age-appropriate scores in their mathematical abilities. In some cases, these adolescents, despite scoring lower in certain areas, demonstrated competencies within the normal range for other mathematical domains. This indicates that a subset of children with ASD can indeed master complex mathematical concepts. One possible explanation for these findings is the variability in cognitive profiles associated with ASD (Mandy et al., 2015), where specific cognitive strengths may help offset challenges in other areas.

The results from the correlation analysis at the group level suggest that while NVIQ abilities are strongly related to mathematical performance in both groups, verbal skills may play a more pronounced role in the mathematical abilities of children with TD. Although we did not detect significant differences in cognitive and linguistic abilities between groups, this does not necessarily imply that these abilities are identical or similar. The weaker correlations observed in the ASD group, particularly between receptive vocabulary and mathematical scores, may still reflect the variability in how children with ASD acquire and process language. Even with age-appropriate receptive vocabulary, children with ASD might exhibit differences in the integration of linguistic abilities due to challenges in pragmatic language use, social communication, or attention. These factors could interfere with forming strong connections between their vocabulary knowledge and other cognitive domains, such as mathematical reasoning, compared to their typically developing peers, and this specific result warrants further research with a larger cohort to better understand these findings.

Across the three subgroups of children with ASD the correlation analysis revealed some interesting differences. In Group 1 (age-matched scores), the unexpected negative correlation between age and procedural thinking suggested that older children may experience more difficulty with procedural tasks, possibly due to differences in cognitive strategies or developmental variations. This finding should be considered with caution as the number of adolescents in the group was small. In Group 2 (uneven performance across tasks), the strong correlations between NVIQ and mathematical performance indicated that children with higher NVIQ tend to perform better in mathematical tasks. However, the uneven performance across tasks suggests that some children may excel in certain domains while struggling in others. This pattern highlights the importance of identifying individual strengths and weaknesses to create targeted interventions that address specific areas of need. For Group 3 (overall low scores), the strong correlations between mathematical scores, procedural thinking, and arithmetic comprehension suggest that despite overall lower performance, children who demonstrate strengths in one area of mathematical abilities tend to perform well across related tasks. This finding suggests that interventions aimed at improving performance in one mathematical domain could have broader benefits, helping to support overall mathematical development in children with lower abilities.

The current study, while insightful, is subject to several limitations that warrant consideration. First, the sample size is relatively modest, comprising 67 participants, with 31 being adolescents with ASD. To enhance the robustness of the findings, future studies should strive to recruit a larger cohort of adolescents with ASD. Moreover, it is important to note that certain subparts of the tasks included only 2–5 items, resulting in relatively low raw scores. When these scores were converted into percentages, the small number of items sometimes led to low percentage values, potentially impacting the overall interpretation of the data. Nevertheless, these scores were still able to indicate the differences between groups. This limitation underscores the need for future studies to incorporate a greater number of items or tasks within each sub-domain to ensure more robust and accurate measurement. Expanding the item pool would help provide a more comprehensive understanding of the abilities being assessed and improve the reliability and generalizability of the findings.

Another limitation of this study was the very large standard deviations observed in the typically developing (TD) group. These wide variations suggest a high level of individual differences within this group, which may have impacted the overall results and reduced the ability to detect more subtle differences between the TD and ASD groups. Future research should consider strategies to account for this variability, such as using larger sample sizes or employing methods that can better handle heterogeneous data, to ensure more accurate and representative comparisons.

Finally, it is important to interpret the lack of observed differences in cognitive and linguistic abilities between groups with caution. Given the sample size and statistical power of this study, the absence of significant differences may reflect a Type II error—where true differences exist but were not detected due to limited sensitivity in the analysis. Consequently, while our findings did not reveal cognitive or linguistic disparities between groups, this does not definitively indicate that these abilities are comparable. Further research with a larger sample and increased statistical power would be beneficial to clarify whether meaningful differences might be present in these domains.

Additionally, a comprehensive intervention study is warranted, delving into the methodologies of learning, teaching, and evaluating mathematical skills in children and adolescents with ASD, tailoring approaches to address their specific challenges.

## Summary and Conclusions

This study identified significant differences in mathematical skills between adolescents with ASD and their typically developing peers. The observed performance differences emphasizes the necessity for further research to inform effective approaches for teaching and assessing mathematical skills in individuals with ASD. Our analysis of procedural thinking, arithmetic comprehension, and algebraic technique contributes to ongoing discussions on the importance of addressing their unique educational needs in mathematics. Moreover, it is noteworthy that considerable variability was evident within the ASD group: while some adolescents in this cohort displayed consistently low scores across all tested mathematical domains, others exhibited mixed performances, demonstrating proficiency in certain mathematical skills while struggling with others. The prevalence of low performance among many adolescents with ASD in our study highlights the significance of early identification and intervention akin to current practices in identifying and treating speech difficulties in young children with ASD. Early detection and targeted intervention strategies tailored to address mathematical challenges hold the potential to enhance the mathematical abilities and overall functioning of adolescents with ASD in the long term.

## Supplementary Information

Below is the link to the electronic supplementary material.

Supplementary file1 (DOCX 16 kb)

Supplementary file2 (DOCX 47 kb)
