# Supplementary material for: Mathematical Proficiency in Adolescents with ASD
Source: J Autism Dev Disord. 2024 Nov 26;56(4):1382–97. doi: 10.1007/s10803-024-06645-3 (PMC12987910; doi:10.1007/s10803-024-06645-3)
Supplement: Supplementary file 1 — Supplementary file1 (DOCX 16 kb) [file 10803_2024_6645_MOESM1_ESM.docx]

APPENDIX A

**The Linguistic tasks Coding Scheme**

*Phoneme Segmentation* - (Schiff, 2004) - Phonological awareness was assessed using the division into phonemes test from the Hadad Center at Bar Ilan University. Participants were presented with words and asked to break them into constituent sounds. The test included six categories, ranging from two to seven phonemes, with three lists per category. Participants were read two words per round (from list 1 and list 2), and if an error occurred, an additional word from list 3 was presented. One point was awarded for each correctly segmented word, with a maximum score of 12 points, converted to percentages for analysis. While not previously administered to children with ASD, the test had been used with children with learning disabilities and attention difficulties.

*Morphological Deduction Test* (Schiff et al., 2011) - This assessment evaluates morphological awareness and is part of the diagnostic system at the Hadad Center at Bar Ilan University. Participants were given fifteen sentences, each with a missing word, and the root of the word provided in parentheses at the end of the sentence. They were required to complete the sentences by selecting a word that matched the given root, applying the appropriate morpho-syntactic pattern. Each correctly completed word earned one point, with a maximum score of 15 points, which was converted to percentages for analysis. Although not previously administered to children with ASD, this test has been used with children with learning disabilities and attention difficulties.

*Definition Task* (Sukenik, 2023) - This task assesses both syntactic and pragmatic skills. Participants were instructed to verbally define a series of 39 words presented individually. Responses were evaluated for syntax and pragmatics (see Appendix 1 for details). Syntactic abilities were assessed and coded using two parameters: syntactic accuracy and sentence construction ability. Syntactic accuracy was scored with one point for each error, so lower scores indicated better accuracy. Sentence construction was scored from zero (single word or no response) to higher points for more complex sentences, with a maximum of 39 points, converted to percentages for analysis.

Pragmatic abilities were evaluated based on the clarity and correctness of responses. Two female judges classified responses as pragmatically correct or incorrect, resolving disagreements through discussion. Participants received one point for each correct response, with a maximum score of 39 points per category, also converted to percentages for analysis. This test has been used previously with children with ASD and effectively identifies syntactic and pragmatic difficulties*.*

*Children's Communication Checklist-2* (Bishop, 1998) - This questionnaire was used to assess pragmatic skills, though it was originally designed for parental administration. Due to Ministry of Education restrictions, the questionnaire was instead distributed to educational staff. The CCC questionnaire, which focuses on evaluating communication skills, consists of 70 items divided into nine areas. Two areas pertain to formal language aspects (speech and syntax), five areas assess pragmatic communication aspects (inconsistent intonation, coherence, stereotypicality in conversation, use in context, and relationship understanding), and two areas evaluate non-linguistic aspects of autistic behavior (social relationships and purpose). Teachers completed the Hebrew version of the CCC questionnaire. Sub-scores were derived from this questionnaire to specifically assess the subjects' pragmatic and syntactic abilities.

*Shemesh* –Shemesh – Naming 100 nouns - (Biran & Friedmann, 2004) - aimed to assess semantics by evaluating the accuracy and fluency of noun identification. Participants were shown pictures one at a time and asked to verbally identify the depicted object. The number of correct responses for each participant was tallied, and errors were categorized based on Biran and Friedmann's research. Categories included immediate correct responses, correct responses after hesitation (more than five seconds), correct responses following an initial incorrect response, and errors made during correction attempts (total incorrect responses during attempts to rectify mistakes). This test has previously been administered to children with ASD and has proven effective in identifying semantic difficulties (Brock, Sukenik, & Friedmann, 2017; Sukenik, 2017).
