# Supplementary material for: Mathematical Proficiency in Adolescents with ASD
Source: J Autism Dev Disord. 2024 Nov 26;56(4):1382–97. doi: 10.1007/s10803-024-06645-3 (PMC12987910; doi:10.1007/s10803-024-06645-3)
Supplement: Supplementary file 2 — Supplementary file2 (DOCX 47 kb) [file 10803_2024_6645_MOESM2_ESM.docx]

**APPENDIX B**

The Mathematics Skills Test Coding Scheme

| Mathematical domains | Mathematical Proficiency | Written/  oral | Example | Item number | Scoring and maximum score | Score type (percent correct / time) |
| --- | --- | --- | --- | --- | --- | --- |
| Mathematical scores | | Written+ verbal | Example of the questions in the following sections | 1 until 22 | 37 points |  |
| Procedural thinking | | Written+ verbal | Example of the questions in the following sections | 17,8b.9,10a,  11.18,19-21 | 23 points | Percent |
| Mastering the basic number combinations | | Verbal | The participant is asked to retrieve basic facts and must orally answer the question within a limited time frame.  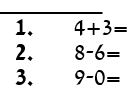 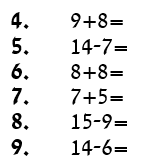 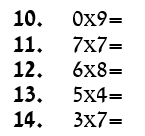 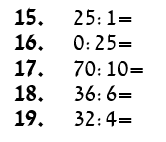 | 18,19,20,21 | 1 point for each section - a total of 8 points | Percent |
|  | Sums to 10-accuracy | Verbal |  | 18a-d | 1/2 point for each section - a total of 2 points | Percent |
|  | Sums to 10- time | Verbal |  |  |  |  |
|  | [Sums to](https://www.ixl.com/math/grade-2/add-one-digit-numbers-sums-to-10) 20- accuracy | Verbal |  | 19a-d | 1/2 point for each section - a total of 2 points | Percent |
|  | [Sums to](https://www.ixl.com/math/grade-2/add-one-digit-numbers-sums-to-10) 20- time | Verbal |  |  |  |  |
|  | With multiplication - accuracy | Verbal |  | 20a-d | 1/2 point for each section - a total of 2 points | Percent |
|  | Multiplication - time | Verbal |  |  |  |  |
|  | Division - accuracy | Verbal |  | 21a-d | 1/2 point for each section - a total of 2 points | Percent |
|  | Division -time | Verbal |  |  |  |  |
|  | Producing a Sequential Counting strategy | Verbal | The participant must count in an ascending sequence up to the number 5,771, and in a descending sequence up to the number 5,399 without mistakes. | 1 a-b | 1 point for each section - a total of 2 points | Percent |
|  | Addition- written algorithm | Written | 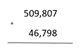 | 2 | 1 point | Percent |
|  | Subtraction- written algorithm | Written | 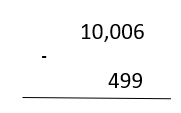 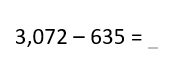 | 3,4,9 | 1 point for each section - a total of 3 points | Percent |
|  | Combining procedures: addition and subtraction | Written | 540 – 405 + 160 = | 1 | 1 point | Percent |
|  | Multiplication-written algorithm | Written  + verbal | 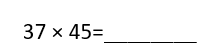 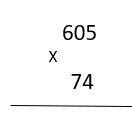 | 5,6;10a,13,22a-b | 1 point for each section - a total of 6 points | Percent |
|  | Long-division | Written | 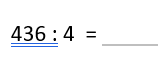 | 7 | 1 point | Percent |
|  | Order of operations | Written | 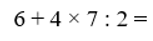 | 8b,11 | 1 point for each section - a total of 2 points | Percent |
| Arithmetic comprehension | | Written  +verbal | Explanation of the questions in the following sections | 9,11,8a,10,12-14,  22 | 10 points | Percent |
|  | Word problems | Written | Explanation of the questions in the following sections | 9,11,8a,10 | 5 points | Percent |
|  | Word problems that require process thinking | Written | The participant must build a correct algorithm according to the details of the question. | 9,11 | 1 point for each section - a total of 2 points | Percent |
|  | Word problems that require metacognitive thinking | Written | The participant is required to have a deep understanding and high-level thinking that requires search and analysis-synthesis, in order to derive meaning since the information is not given explicitly. | 8a,10a-b | 1 point for each section - a total of 3 points | Percent |
|  | Numerical insight | Written  +verbal | The participant is required to answer the question when he relies on mathematical insights of various kinds | 12,13,14,22a-b | 1 point for each section - a total of 5 points | Percent |
| Algebraic Technique | | Written |  | 15,16,17 | 4 points |  |
|  | Procedure | Written | 3x – 1 = 2x +7 | 15,16 | 1 point for each section - a total of 2 points | Percent |
|  | Solving a word problem with unknowns | Written | The participant is required to identify an inequality that corresponds to a verbal representation of the question, this section requires process thinking, integration between concepts, matching a mathematical representation to a verbal representation and algebraic and numerical insight. | 17a-b | 1 point for each section - a total of 2 points | Percent |
